# Supplementary material for: Exploring risk and protective factors which distinguish suicidal and self-harm behaviours from suicidal and self-harm ideation in young people: A systematic review
Source: PLoS One. 2025 Sep 24;20(9):e0326381. doi: 10.1371/journal.pone.0326381 (PMC12459848; doi:10.1371/journal.pone.0326381)
Supplement: S1 Table — (DOCX) [file pone.0326381.s001.docx]

| **Table S1. Quality assessment tool and scoring guide** | | | | |
| --- | --- | --- | --- | --- |
| **Score**  **Criteria** | 0 | 1 | 2 | 3 |
| **Design** | Cross-sectional | Longitudinal | Experimental |  |
| **Power calculation conducted for sample size** | No power calculation. | Power calculation conducted, but sample size not met. | Power calculation conducted and sample size requirements met. |  |
| **Self-harm and suicidal ideation and behaviours measure** | No mention of how self-harm ideation/ behaviour and suicide ideation/ behaviour was assessed. | Non-validated scale or other means of self-report *(e.g. single question).* Measure is invalid or unreliable. | Suicide/ self-harm items from a validated diagnostic/ mood rating scale *(e.g. BDI).* | Clinical interview; validated self-harm, suicide scale *(e.g., SSI)*. |
| **Risk/ protective factor measure** | No mention of how risk/ protective factors were assessed. | Non-validated scale or other means of self-report *(e.g. single question).* Measure is invalid or unreliable. | Validated scale of some, but not all predictor measures. | Validated scale of all predictor measures. |
| **Confounding variables**  *To what extent has there been an attempt to account for confounding variables* | No attempt to account for potential confounding variables in recruitment or analysis. | Accounts for basic confounding variables either during recruitment or analysis.  *e.g. Age, Gender* | Accounts for additional confounding variables either during recruitment or analysis.  *e.g. Mental illness, substance abuse, childhood trauma.* |  |

Note. The characteristics assessed were guided by an methodological quality assessment instrument, tailored for the current review (see O’Connor et al. [1]).

1. O’Connor DB, Ferguson E, Green JA, O’Carroll RE, O’Connor RC. Cortisol levels and suicidal behavior: A meta-analysis. Psychoneuroendocrinology. 2016; 63: 370-379.
