# Supplementary material for: Exploring risk and protective factors which distinguish suicidal and self-harm behaviours from suicidal and self-harm ideation in young people: A systematic review
Source: PLoS One. 2025 Sep 24;20(9):e0326381. doi: 10.1371/journal.pone.0326381 (PMC12459848; doi:10.1371/journal.pone.0326381)
Supplement: S2 Table — (DOCX) [file pone.0326381.s002.docx]

**Table S2. Quality assessment for suicidal ideation and suicide behaviours.**

| **Author and date** | **Research design** | **Power calculation conducted for sample size** | **Suicidal ideation and behaviours measure** | **Risk/ protective factor measure** | **Confounding variables** | **Total score** |
| --- | --- | --- | --- | --- | --- | --- |
| Ahmadboukani et al. [116] | 0 | 2 | 3 | 3 | 0 | 8 |
| Ai et al. [66] | 0 | 0 | 3 | 3 | 0 | 6 |
| Alarcón et al. [67] | 0 | 0 | 3 | 3 | 2 | 8 |
| Alqueza et al. [43] | 0 | 0 | 2 | 3 | 2 | 7 |
| Auerbach et al. [123] | 0 | 0 | 3 | 3 | 2 | 8 |
| Berny & Tanner-Smith [60] | 0 | 0 | 3 | 3 | 2 | 8 |
| Berona et al. [125] | 1 | 0 | 1 | 3 | 2 | 7 |
| Burke et al. [126] | 0 | 0 | 1 | 3 | 0 | 4 |
| Calear et al. [127] | 0 | 0 | 1 | 3 | 2 | 6 |
| Castaldo et al. [128] | 0 | 0 | 3 | 3 | 2 | 8 |
| Chu et al. [34] | 2 | 0 | 3 | 3 | 2 | 10 |
| Claudius & Axeen [35] | 1 | 0 | 3 | 3 | 2 | 9 |
| Commisso et al. [132] | 1 | 0 | 3 | 3 | 2 | 9 |
| Cramer et al. [133] | 0 | 0 | 3 | 3 | 1 | 7 |
| Cruz et al. [134] | 0 | 0 | 1 | 3 | 0 | 4 |
| Dadras & Wang [40] | 0 | 0 | 1 | 1 | 1 | 3 |
| Dean-Boucher et al. [88] | 0 | 0 | 3 | 1 | 2 | 6 |
| Delfabbro et al. [61] | 0 | 0 | 1 | 3 | 0 | 4 |
| De Luca et al. [96] | 1 | 0 | 1 | 1 | 2 | 5 |
| Eisenlohr-Moul et al. [38] | 1 | 0 | 2 | 3 | 2 | 8 |
| Fang [135] | 1 | 0 | 1 | 1 | 2 | 5 |
| Fekadu et al. [79] | 0 | 0 | 3 | 2 | 1 | 6 |
| Florez et al. [41] | 0 | 0 | 3 | 3 | 1 | 7 |
| Gatta et al. [42] | 0 | 0 | 3 | 3 | 0 | 6 |
| Georgiades et al. [45] | 0 | 0 | 1 | 2 | 2 | 5 |
| Glenn et al. [137] | 0 | 0 | 3 | 3 | 2 | 8 |
| Glenn et al. [47] | 1 | 0 | 3 | 2 | 2 | 8 |
| Heelis et al. [138] | 0 | 0 | 3 | 3 | 0 | 6 |
| Hielscher et al. [139] | 1 | 0 | 3 | 3 | 1 | 8 |
| Hong et al. [44] | 0 | 0 | 3 | 3 | 2 | 8 |
| Ivanich et al. [55] | 0 | 0 | 1 | 1 | 0 | 2 |
| Jenkins et al. [142] | 0 | 0 | 2 | 3 | 0 | 5 |
| Khoubaeva et al. [63] | 0 | 0 | 3 | 3 | 1 | 7 |
| Kim et al. [56] | 0 | 0 | 1 | 1 | 2 | 4 |
| Kwon et al. [144] | 0 | 0 | 1 | 3 | 0 | 4 |
| Kwon et al. [31] | 0 | 0 | 0 | 1 | 0 | 1 |
| Kyron et al. [49] | 0 | 0 | 1 | 2 | 1 | 4 |
| Lear et al. [52] | 0 | 0 | 1 | 2 | 0 | 3 |
| Li et al. [74] | 0 | 0 | 1 | 1 | 1 | 3 |
| Liu et al. [109] | 0 | 2 | 3 | 1 | 0 | 6 |
| Liu et al. [146] | 0 | 0 | 2 | 1 | 2 | 5 |
| Liu et al. [64] | 0 | 0 | 3 | 3 | 2 | 8 |
| Macrynikola et al. [85] | 0 | 0 | 1 | 2 | 1 | 4 |
| Marengo et al. [36] | 1 | 0 | 3 | 2 | 2 | 8 |
| Mars et al. [11] | 1 | 0 | 1 | 2 | 1 | 5 |
| Mars et al. [58] | 0 | 0 | 1 | 2 | 1 | 4 |
| Masi et al. [50] | 0 | 0 | 3 | 2 | 0 | 5 |
| May et al. [75] | 0 | 0 | 1 | 1 | 0 | 2 |
| McCallum et al. [78] | 0 | 0 | 1 | 3 | 0 | 4 |
| McKay et al. [32] | 0 | 0 | 0 | 0 | 1 | 1 |
| Melhem et al. [92] | 0 | 0 | 3 | 3 | 2 | 8 |
| Mortier et al. [151] | 0 | 0 | 3 | 1 | 1 | 5 |
| Musci et al. [152] | 1 | 0 | 3 | 1 | 1 | 6 |
| Nestor et al. [73] | 1 | 0 | 1 | 2 | 2 | 6 |
| Okado et al. [33] | 0 | 0 | 1 | 1 | 0 | 2 |
| Ozger et al. [80] | 0 | 0 | 3 | 3 | 0 | 6 |
| Paul et al. [71] | 0 | 0 | 3 | 1 | 2 | 6 |
| Plener et al. [84] | 0 | 0 | 3 | 3 | 0 | 6 |
| Puangsri & Ninla-aesong [93] | 0 | 0 | 3 | 3 | 2 | 8 |
| Quevedo et al. [90] | 0 | 0 | 3 | 3 | 2 | 8 |
| Rajappa et al. [156] | 0 | 0 | 3 | 3 | 0 | 6 |
| Ren et al. [48] | 0 | 0 | 1 | 3 | 0 | 4 |
| Rengasamy et al. [95] | 0 | 0 | 3 | 3 | 2 | 8 |
| Rengasamy et al. [94] | 1 | 0 | 3 | 3 | 2 | 9 |
| Rivers et al. [86] | 0 | 0 | 2 | 2 | 0 | 4 |
| Robinson et al. [156] | 0 | 0 | 3 | 3 | 1 | 7 |
| Rooney et al. [76] | 0 | 0 | 1 | 1 | 1 | 3 |
| Saffer et al. [83] | 0 | 0 | 1 | 3 | 2 | 6 |
| Santana et al. [54] | 0 | 0 | 3 | 3 | 2 | 8 |
| Schlagbaum et al. [72] | 0 | 0 | 3 | 3 | 2 | 8 |
| Scott et al. [39] | 1 | 0 | 3 | 3 | 2 | 9 |
| Sewall et al. [81] | 1 | 0 | 3 | 3 | 2 | 9 |
| Soylu et al. [82] | 0 | 0 | 1 | 3 | 0 | 4 |
| Stack [59] | 0 | 0 | 1 | 1 | 2 | 4 |
| Stange et al. [91] | 0 | 0 | 3 | 3 | 1 | 7 |
| Stewart et al. [12] | 0 | 0 | 2 | 3 | 0 | 5 |
| Stewart et al. [163] | 0 | 0 | 2 | 3 | 2 | 7 |
| Stewart et al. [68] | 0 | 0 | 2 | 3 | 2 | 7 |
| Taliaferro & Muehlenkamp [77] | 0 | 0 | 1 | 1 | 1 | 3 |
| Tanner et al. [69] | 0 | 0 | 1 | 3 | 1 | 5 |
| Valderrama et al. [65] | 0 | 0 | 3 | 3 | 1 | 7 |
| Vélez-Grau et al. [53] | 0 | 0 | 1 | 1 | 2 | 4 |
| Vergara et al. [51] | 0 | 0 | 2 | 3 | 1 | 6 |
| Voss et al. [164] | 0 | 0 | 3 | 1 | 1 | 5 |
| Wang et al. [62] | 0 | 0 | 1 | 1 | 2 | 4 |
| Wang et al. [165] | 0 | 0 | 1 | 2 | 0 | 3 |
| Wang et al. [57] | 0 | 0 | 1 | 1 | 2 | 4 |
| Wetherall et al. [70] | 0 | 0 | 1 | 3 | 2 | 6 |
| Yan et al. [46] | 0 | 0 | 1 | 3 | 0 | 4 |
| Yang et al. [87] | 0 | 0 | 3 | 3 | 2 | 8 |
| You & Lin [167] | 1 | 0 | 1 | 1 | 2 | 5 |
| Zhong et al. [89] | 0 | 0 | 3 | 2 | 2 | 7 |
