# Supplementary material for: Exploring risk and protective factors which distinguish suicidal and self-harm behaviours from suicidal and self-harm ideation in young people: A systematic review
Source: PLoS One. 2025 Sep 24;20(9):e0326381. doi: 10.1371/journal.pone.0326381 (PMC12459848; doi:10.1371/journal.pone.0326381)
Supplement: S3 Table — (DOCX) [file pone.0326381.s003.docx]

| **Author and date** | **Research design** | **Power calculation conducted for sample size** | **Self-harm ideation and behaviours measure** | **Risk/ protective factor measure** | **Confounding variables** | **Total score** |
| --- | --- | --- | --- | --- | --- | --- |
| Del Carpio et al. [97] | 1 | 0 | 2 | 3 | 3 | 9 |
| García-Neito et al. [166] | 0 | 0 | 2 | 3 | 0 | 5 |
| Jiang et al. [170] | 0 | 0 | 1 | 3 | 1 | 5 |
| McMahon et al. [171] | 0 | 0 | 1 | 1 | 0 | 2 |
| Madge et al. [99] | 0 | 0 | 1 | 2 | 2 | 5 |
| O’Connor et al. [24] | 0 | 0 | 1 | 2 | 2 | 5 |
| Saarijärvi et al. [98] | 1 | 0 | 3 | 3 | 0 | 7 |

**Table S3. Quality assessment for self-harm ideation and self-harm behaviours.**
