# Supplementary material for: Exploring risk and protective factors which distinguish suicidal and self-harm behaviours from suicidal and self-harm ideation in young people: A systematic review
Source: PLoS One. 2025 Sep 24;20(9):e0326381. doi: 10.1371/journal.pone.0326381 (PMC12459848; doi:10.1371/journal.pone.0326381)
Supplement: Attachment — Submitted filename: plos review report.pdf [file pone.0326381.s005.pdf]

Dear Editor,

Dear Editor, I would like to express my gratitude to you and the Associate Editor for allowing me to review the manuscript **"Risk and Protective Factors that distinguish those who have thoughts of self-harm or suicide from those who act on them: A Systematic Review in young people".** I approached the article with great enthusiasm and believe that it offers valuable insights into risk factors of suicidal thoughts of self-harm with a focus on critical thinking. The study's findings are quite revealing among young people. It indicates that risk factors that distinguished self-harm behavior from thoughts of self-harm included being female, exposure to self-harm/suicide, and impulsivity. However, to enhance the manuscript, the authors should have considered certain considerations, which would have made it more appealing to international readers.

General comment: Many typographical and grammatical errors should be attended to. Therefore, the writing needs language, grammar, and punctuation revision.

Regarding the specific aspects of the article:

Dear authors, Thank you for conducting such a valuable study. I have reviewed your manuscript thoroughly and come up with the following comments section by section.

Title: Risk and protective factors that distinguish those who have thoughts of self-harm or suicide from those who act on them: A systematic review in young people.

Abstract: Please clarify the abstract, particularly regarding the results. It is currently vague and lacks a concise summary, which may discourage researchers from reading it if published as is. Therefore, please rewrite the entire abstract.

Organize the abstract into the following sections according to the journal guidelines: background, methods, results, and conclusions.

<u>Introduction</u>

The literature review in the manuscript is comprehensive and timely. I recommend that the authors incorporate specific evidence and references from the literature to bolster their arguments, enhancing the credibility of their findings and offering readers a broader perspective on the topic.

Chosen methods, results, and data interpretation are within the higher-quality papers; however, they are missing originality: If there are plenty of studies according to your findings, **why is your study unique?** Several studies, including meta-analyses, have examined protective and risk factors associated with suicidal behaviors, such as thoughts, ideation, attempts, and completed suicide. Therefore, the authors need to justify the necessity of this study more effectively.

Research question

What risk and protective factors distinguish young people (aged 13-25 years) who have thoughts of self-harm or suicide from those who act on them? What age classification for young people (13-25 years) did the WHO establish?

How did you see the age category of children and adolescents according to WHO? This needs clear justification.

The methodology section lacks clarity on the criteria for including and screening literature. The authors should explicitly state these criteria and detail how they selected the articles for their review.

Please clarify your inclusion and screening criteria, as they are currently vague and not briefly summarized, which may deter researchers from reading your work if published in this manner.

Did you gather data on suicidal ideation and attempts over the past month, 12 months, or a lifetime, including the instruments used? Additionally, did you assess self-harm and suicidal ideation and behaviors together as a single outcome (suicidality)? Please clarify.

Results

Generally, your results part is not written as it fits scientific journals. Rewrite it, including descriptive parts.

Why did you exclude studies examining risk or protective factors for self-harm ideation and self-harm behavior or suicidal ideation and suicide attempts? And also, what about qualitative studies ?

What were the methodological quality assessment scores for studies on suicidal ideation and behaviors, which encompass all aspects from thoughts to actions? How did you handle data quality management?

Discussion

This review aimed to synthesize findings from existing research that have examined risk or protective factors in young people that distinguish between those who have thoughts of self-harm or suicide and those who act on their thoughts. Therefore, what was the outcome? either risk protective / both? If the outcome was both a risk and protective factor, address each separately.

Your discussion is too shallow, so discuss it in more depth.

Discuss your study with a systematic review and meta-analysis. Are there differences? If so, why? Compare your independent predictors with other studies. Discuss in more depth.

You compared your study to a single cross-sectional study in the discussion section. Is that appropriate? Please revise it.

Generally, your comparisons in the discussion part were shallow. So revise it and put your impression/insight based on your findings.

Thank you once again for the opportunity to review this work.
