# Supplementary material for: Exploring risk and protective factors which distinguish suicidal and self-harm behaviours from suicidal and self-harm ideation in young people: A systematic review
Source: PLoS One. 2025 Sep 24;20(9):e0326381. doi: 10.1371/journal.pone.0326381 (PMC12459848; doi:10.1371/journal.pone.0326381)
Supplement: Attachment — Submitted filename: PLoS One Response to Reviewers_ME_roc2.docx [file pone.0326381.s007.docx]

**Date:** May 15, 2025

**Ref:** PONE-D-24-41487

**Associate Editor:** Dr Alemayehu Molla Wollie

**Journal:** *Plos One*

**Title:** *Risk and protective factors that distinguish those who have thoughts of self-harm or suicide from those who act on them: A systematic review in young people.*

Dear Dr Alemayehu Molla Wollie,

We are extremely grateful for the suggestions that have helped us to improve our manuscript considerably. We have revised our manuscript in consideration of the comments and suggestions provided and have detailed changes to the manuscript below. Specific changes to the manuscript have been submitted using tracked changes and are presented in this letter in red font.

**Comments from Editor:**

Editor: *An abstract may need subsections (background, objective, methodology, result, and discussion).*

**Response:** Thank you for your comment. We couldn’t see any guidance for these subsections on the guidelines for authors and also couldn’t see Abstract subsections included on previous published *Plos One* papers (e.g., Bayliss et al., 2022). Nevertheless, we are happy to include these and have now provided subsections in the abstract.

Editor: *Clarify the reason why the term ‘protective factors’ has been included in the title since you didn’t find any single study regarding this, as you mentioned in the abstract.*

**Response:** The term ‘protective factors’ has been included in the title because, although there were only a few studies, we did find evidence that some protective factors distinguished between suicide attempts and thoughts of suicide in the current review. Protective factors that distinguished suicide attempts from suicidal thoughts included parental factors (e.g., parental connectedness) and greater academic achievement (Taliaferro & Muehlenkamp, 2014; Okado et al., 2021; Wang et al., 2023). However, we found no studies of protective factors that distinguished between self-harm thoughts and self-harm behaviours. In summary, therefore, we think it is important to include this in the title as this is part of the original aim of the review. Indeed, the limited evidence of protective factors is a key take-home message of the review.

Editor: *Introduction, Paragraph 1, “Despite this prevalence, little is known regarding the distinct risk and protective factors that distinguish young people who have thoughts of self-harm or suicide from those who act on their thoughts. It is, therefore, important to identify factors involved in this transition, with the intention to inform future preventative and treatment efforts.” I recommend authors shift this statement to the last paragraph of the introduction section. Even I can see a similar concept from the last paragraph of the introduction.*

**Response:** Thank you for your comment. We agree that this statement would fit better in the last paragraph of the introduction and have therefore shifted this statement to the last paragraph of the introduction. In addition, we have also added a sentence at the end of the first paragraph so that it transitions more smoothly to the next paragraph and gives a clear indication of what the review is about. Specific changes are as follows:

Introduction, *first paragraph:* “It is, therefore, important to better understand the risk and protective factors associated with whether young people with thoughts of self-harm or suicide will engage in self-harm or suicidal behaviours”.

Editor: *Please include some evidence regarding the eligibility criteria based on study types (qualitative, quantitative, mixed..). In addition, for the benefit of the readers, it is good to indicate some reasons for restricting studies based on years of publication.*

**Response:** Thank you for your comment. The relevant responses are included within distinct subheadings below:

*Study types*:

Regarding study types, our review had no restrictions on study type (qualitative, quantitative, mixed methods), however no studies were retrieved that met the inclusion criteria for qualitative or mixed methods studies, hence only quantitative studies were included. In our narrative synthesis section, we noted that “No qualitative studies met the inclusion criteria” but we agree this needs to be made clearer in the manuscript and have subsequently made the following changes to our manuscript:

Methods, *Inclusion criteria*: “For inclusion in the review, studies had to: (a) be a primary empirical paper of any study type (e.g., quantitative, qualitative, mixed methods)”.

*Results*: “All studies that met the inclusion criteria and thus were included in the review were quantitative”.

Narrative synthesis, *Study characteristics*: *“*All studies were quantitative;*”.*

*Years of publication*:

Regarding restricting studies based on years of publication, the start date of 2011 was chosen based on the publication of the updated IPTS (Van Orden et al., 2010) and emergence of the IMV model (O’Connor, 2011), both ideation-to-action frameworks. Here, we envisaged a lack of research using the ideation-to-action framework prior to this date. We have touched upon this in our limitations section “In addition, the search was conducted between 2011-2024, therefore relevant studies published before 2011 would not have been retrieved (e.g., [102]). Most studies examining the “ideation-to-action” framework, however, were likely published after 2011 and this review synthesizes contemporary evidence on this framework.” However, we have now provided further detail in our manuscript to make this clear:

Methods, *Search strategy:* “The start date of 2011 was chosen based on the publication of the updated IPTS (Van Orden et al., 2010) and emergence of the IMV model (O’Connor, 2011), both ideation-to-action frameworks”.

Editor: *No evidence regarding risk or protective factors from your searching terms.*

**Response:** Thank you for your comment. You make a good point. However, we decided not to include specific search terms for ‘risk’ or ‘protective’ factors in our database search given that factors associated with suicide are complex and hence not all studies included in the review would necessarily include these terms in the title or abstract. Instead, studies may include other general terms (e.g., key differences, social, neural, biological and psychological factors) or more specific terms (i.e., the specific risk factors investigated). In response to this comment, we have reviewed the title and abstracts of included studies and while several studies do include these terms, several do not despite being focused on risk or protective factors (e.g., Auerbach et al., 2015; Fekadu et al., 2016). Therefore, some studies would have been missed. Hence, we believe that our approach is more inclusive and feel that it is better to examine whether studies focused on risk or protective factors in the screening process instead.

Editor: *I am not sure your article screening approach is appropriate since only first authors conducted overall screening. I hope you have evidence on how authors can screen and extract articles using Covidence or other software together.*

**Response:** Thank you for your comment. We are slightly unsure on what the editor means regarding the first author conducting overall screening. We used Endnote for our screening process, where retrieved articles from the search were then imported to Excel. We removed duplicates both electronically using Endnote and manually when articles were exported into an Excel document. We then screened title and abstracts of studies based on the inclusion criteria and subsequently screened the remaining studies by their full-text. To check for accuracy of the screening process, a second assessor screened a random 10% sample of title and abstracts and a random 20% sample of full-text papers. Both reviewers (the first author and the co-author) reached excellent agreement for title and abstract screening (Κappa (K) = 0.81) and substantial agreement for full-text screening (K = 0.65). All disagreements were resolved through discussion. We provided details of this process in the manuscript (see *Methods, screening,* page 7). We also provided details regarding the software used and the deduplication process in the Results section, but have now moved this to the methods section (*Methods, screening,* page 7). Specific changes are as follows:

*Methods, screening,* page 7: “Search results from each database were exported to Endnote. Duplicates were removed electronically in Endnote and manually when articles were exported into an Excel document. The first author assessed the retrieved studies’ titles and abstracts for inclusion. In cases where it was unclear if a study met the inclusion criteria, the study was retained to be screened by its full text. To check for accuracy of the screening process, a second assessor screened a random 10% sample of title and abstracts and a random 20% sample of full-text papers. The two reviewers reached excellent agreement for title and abstract screening (Κappa (K) = 0.81) and substantial agreement for full-text screening (K = 0.65). Any disagreements were resolved through discussion”.

Editor: *I am not sure how authors extracted results of qualitative and /or quantitative studies if they considered both.*

**Response:** Thank you for your comment. Our inclusion criteria included both quantitative and qualitative studies, however no qualitative studies were found that met our inclusion criteria. For the included quantitative studies, we extracted data based on author and date of publication, sample type and demographics, study design, country, risk and protective factors examined, outcome measure, significant risk or protective factors found and study results We provided details of this process in the manuscript (see *Methods, data extraction,* page 7).

**Comments from Reviewer 1:**

Reviewer: *I would like to express my gratitude to you and the Associate Editor for allowing me to review the manuscript “Risk and Protective Factors that distinguish those who have thoughts of self-harm or suicide from those who act on them: A Systematic Review in young people”. I approached the article with great enthusiasm and believe that it offers valuable insights into risk factors of suicidal thoughts of self-harm with a focus on critical thinking. The study's findings are quite revealing among young people. It indicates that risk factors that distinguished self-harm behavior from thoughts of self-harm included being female, exposure to self-harm/suicide, and impulsivity. However, to enhance the manuscript, the authors should have considered certain considerations, which would have made it more appealing to international readers.*

**Response:** Thank you for your enthusiasm and positive comments regarding our manuscript. Thank you also for your constructive comments. We appreciate the time taken to help us improve our manuscript.

Reviewer: *Many typographical and grammatical errors should be attended to. Therefore, the writing needs language, grammar, and punctuation revision.*

**Response:** Thank you for your comment. We have thoroughly reviewed our manuscript for any typographical or grammatical errors and have attended to any errors found. We have revised our manuscript based on language, grammar and punctuation.

Reviewer: Dear authors, Thank you for conducting such a valuable study. I have reviewed your manuscript thoroughly and come up with the following comments section by section.

**Response:** Thank you for your positive comments regarding our manuscript.

Reviewer: *Abstract: Please clarify the abstract, particularly regarding the results. It is currently vague and lacks a concise summary, which may discourage researchers from reading it if published as is. Therefore, please rewrite the entire abstract.*

*Organize the abstract into the following sections according to the journal guidelines: background, methods, results, and conclusions.*

**Response:** Thank you for your comments. We agree we could have been clearer in our abstract and have revised it in line with your suggestions. We have now also organized our abstract into subsections as recommended.

Reviewer: *Introduction: The literature review in the manuscript is comprehensive and timely. I recommend that the authors incorporate specific evidence and references from the literature to bolster their arguments, enhancing the credibility of their findings and offering readers a broader perspective on the topic. Chosen methods, results, and data interpretation are within the higher-quality papers; however, they are missing originality: If there are plenty of studies according to your findings, why is your study unique? Several studies, including meta-analyses, have examined protective and risk factors associated with suicidal behaviors, such as thoughts, ideation, attempts, and completed suicide. Therefore, the authors need to justify the necessity of this study more effectively.*

**Response:** Thank you for your comment. We have revised our manuscript to include more specific evidence and references to bolster our arguments and to enhance the credibility of our findings.

Notably, many common risk factors for suicide identified in the literature have not distinguished between risk factors for suicidal ideation and risk factors involved in the transition from suicidal ideation to suicide attempts (see May & Klonsky, 2016). In this regard, evidence suggests that many previously identified risk factors for suicide (e.g., depression, hopelessness) are risk factors for suicidal ideation, but not attempts and/or do not significantly distinguish between those with thoughts of suicide and those who attempt suicide (e.g., Mars et al., 2019; Stewart et al., 2017; Qiu et al., 2017). By identifying specific risk and protective factors that increase/ decrease the risk someone with suicidal thoughts may or may not act on their thoughts, we can better inform preventative efforts by targeting the key risk and protective factors involved in this transition (Klonsky & May, 2014).

While reviews and primary studies in adolescents or young people have identified risk and protective factors for one or more of the outcomes including suicidal ideation, suicidal attempts, self-harm ideation and self-harm (e.g., Ati et al., 2021), no reviews have examined risk/ protective factors that distinguish suicide attempts from suicidal ideation or self-harm behaviour from self-harm ideation or factors involved in the transition from suicidal ideation to suicide attempts or self-harm ideation to self-harm attempts. Hence, we are interested in the risk or protective factors that explain *why* an individual with suicidal thoughts may or may not attempt suicide, as well as *why* an individual with self-harm thoughts may or may not engage in self-harm behaviour.

Therefore, our first review is novel as it is the first to synthesise risk and protective factors that distinguish suicide attempts from suicidal thoughts, and self-harm behaviours from self-harm thoughts. This review will provide an important touchstone by synthesising the most up-to-date evidence of these risk and protective factors to guide future research, identify knowledge gaps and inform preventative/ intervention efforts in young people based on key risk/ protective factors identified (e.g., how best to intervene).

We have aimed to more effectively justify the originality and necessity of our study. Specific changes are as follows:

*Introduction, last paragraph*: “Thus far, no reviews have synthesised existing work on the distinct risk and protective factors that distinguish young people who have thoughts of self-harm or suicide from those who act on their thoughts. It is, therefore, important to identify key risk and protective factors involved in this transition. The current review addresses these existing limitations by being the first to synthesise the distinct risk or protective factors which distinguish suicide attempts from suicidal ideation and distinguish self-harm behaviours from self-harm ideation in young people. This comprehensive review provides an important touchstone, synthesising the most up-to-date evidence on the ideation-to-framework in young people, which can be utilised to guide future research, highlight knowledge gaps, and inform future preventative and treatment efforts.”

Reviewer: *Research question: What risk and protective factors distinguish young people (aged 13-25 years) who have thoughts of self-harm or suicide from those who act on them? What age classification for young people (13-25 years) did the WHO establish? How did you see the age category of children and adolescents according to WHO? This needs clear justification. The methodology section lacks clarity on the criteria for including and screening literature.*

*The authors should explicitly state these criteria and detail how they selected the articles for their review. Please clarify your inclusion and screening criteria, as they are currently vague and not briefly summarized, which may deter researchers from reading your work if published in this manner. Did you gather data on suicidal ideation and attempts over the past month, 12 months, or a lifetime, including the instruments used? Additionally, did you assess self-harm and suicidal ideation and behaviors together as a single outcome (suicidality)? Please clarify.*

**Response:** Thank you for your comments. The relevant responses are included within distinct subheadings below:

*Classifying the age range for young people*:

Regarding the age range, there is no universally agreed international definition of young people. Hence, the term ‘youth’ or ‘young people’ can vary depending on the context, with some organizations using different age ranges.

The World Health Organization (WHO) defines young people as individuals aged 10 to 24 years old, which encompasses both adolescents (10-19 years) and youth (15-24 years). In data provided by The Office for National Statistics (ONS) in the UK, the ONS classifies young people as 13-29 years. Some sources classify teenagers as aged 13-19 years. In addition, the upper age limit used to define adolescence in studies varies between 18 years and 25 years (see Hawton et al., 2012). Hence, we chose the age range to classify young people based on various definitions.

We were also mindful that rates of suicide attempts are rare below age 13 years, whereby suicide incidence typically increases with age (Office for National Statistics, 2024; see Rodway et al., 2020). Research suggests that this increase may be partly explained by developmental trajectories of key risk factors, such as onset of psychopathology (Shaffer et al., 1996). So synthesising all of these considerations we felt that classifying young people as those aged 13-25 years old was the most appropriate approach for this review. To reflect this we have added the following to the manuscript:

*Introduction,* Methods: “While there is no universally agreed age range for young people, based on various definitions and because suicide attempts are rare before the teenage years (Office for National Statistics, 2024) and as adolescence is thought to extend until the mid-twenties (e.g., World Health Organisation, 2024), we have classified young people as those aged from 13 to 25 years”.

*Inclusion and exclusion criteria*:

Regarding our inclusion and screening criteria, for inclusion in the review, studies had to: (a) be a peer-reviewed primary empirical paper of any study type (e.g., quantitative, qualitative, mixed methods); (b) be available in English language; (c) include a sample of young people aged between 13-25 years or include a sample with a broader age range than 13-25 years, but where the *M_age_* of participants fell into this range; (d) examine risk or protective factors that distinguished suicide attempts from suicidal ideation or self-harm behaviour from self-harm ideation; and (e) include at least two distinct groups (e.g., a suicidal ideation group with no previous history of suicide attempts vs a suicide attempt group or a self-harm ideation group with no previous history of self-harm behaviour vs a self-harm group) or track the transition from suicidal ideation at baseline (with no prior history of suicide attempts) to suicide attempts or self-harm ideation at baseline (with no prior history of self-harm) to self-harm behaviour over time.

Conversely, studies were excluded if they: (a) were not a peer-reviewed primary empirical paper (e.g., a review article, commentary); (b) were not available in English language (c) did not include a sample of young people within the age range of 13-25 years or where the majority of participants fall into this range; (d) did not examine risk or protective factors that distinguished suicide attempts from suicidal ideation or self-harm behaviour from self-harm ideation and (e) did not examine two distinct groups (e.g., a suicidal ideation group with no previous history of suicide attempts vs a suicide attempt group or a self-harm ideation group with no previous history of self-harm behaviour vs a self-harm group) or track the transition from ideation to behaviours over time. We also excluded studies where the suicidal or self-harm ideation group had a lifetime history of suicidal or self-harm behaviours because the risk and protective factors that distinguish between people with current suicidal or self-harm ideation and a lifetime history of behaviours, and suicidal or self-harm ideation and no history of suicide attempts are likely to differ (see *Methods, inclusion and exclusion criteria,* page 6-7).

*Data on suicidal ideation and attempts over the past month, 12 months, or a lifetime, including the instruments used*:

We gathered data on measures used in individual studies and whether studies measured current suicidal ideation and behaviours, past month, past 6 month, 12-month etc. or lifetime suicidal ideation and behaviours (where provided) and have provided example items documenting this e.g., “Have you attempted to take your own life in the past 12 months?”. These are reported in the suicide measure column in Table 2. We have also reviewed measures included to determine whether more information can be provided on the recency of suicidal ideation and behaviours. As can be seen in Table 2, there was significant heterogeneity regarding the instruments used and the timeframe the instruments covered. We updated our limitations section to include this. Specific changes are as follows:

*Limitations of the review and future directions: “*Furthermore, there was considerable heterogeneity of variables, including measurement of risk and protective factors, outcome variables and the measure timescales (e.g., present, lifetime). Therefore, it was not deemed appropriate to conduct a meta-analysis”.

There were no clear patterns identified between the significance of risk and protective factors in distinguishing suicide attempts from suicidal ideation and self-harm behaviours from self-harm ideation, and the timing of measurement of self-harm and suicide.

*Assessing self-harm and suicidal ideation and behaviors together as a single outcome (suicidality)*:

We did not assess self-harm and suicidal ideation and behaviours together as a single outcome as the aim of our review was to understand the risk/ protective factors that distinguished suicide attempts from suicidal ideation or self-harm behaviours from self-harm ideation. This is noted as a reason for exclusion in the results section and PRISMA diagram in our manuscript. Studies that did examine suicidality as a single outcome were excluded because when ideation and behaviours are examined together, they do not allow us to identify distinct risk or protective factors for experiencing self-harm/ suicidal ideation and distinct risk or protective factors for why people experiencing thoughts of self-harm or suicide act on their thoughts.

Reviewer: *Results: Generally, your results part is not written as it fits scientific journals. Rewrite it, including descriptive parts. Why did you exclude studies examining risk or protective factors for self-harm ideation and selfharm behavior or suicidal ideation and suicide attempts? And also, what about qualitative studies ? What were the methodological* *quality assessment scores for studies on suicidal ideation and behaviors, which encompass all aspects from thoughts to actions? How did you handle data quality management?*

**Response:** Thank you for your comments. In response to your comments, we have edited the results section and the manuscript to ensure it meets the scientific guidelines of PloS One. We excluded studies that examined risk or protective factors for self-harm ideation and/or self-harm behaviour or suicidal ideation and/ or suicide attempts only if studies did not examine risk or protective factors distinguishing between ideation and behaviours (i.e., they need to examine at least two distinct groups (e.g., a suicidal ideation group with no previous history of suicide attempts vs a suicide attempt group or a self-harm ideation group with no previous history of self-harm behaviour vs a self-harm group) or had examined risk/ protective factors involved in the transition from ideation to behaviour. This is because our review is guided by an ideation-to-action framework (see Klonsky & May, 2014), hence studies that do not examine risk/ protective factors distinguishing suicide attempts from suicidal ideation or self-harm behaviour from self-harm ideation (i.e., comparing an ideation and behaviour group) or examining risk/ protective factors involved in the transition from ideation to behaviours are not relevant to the aim of our review.

Our inclusion criteria included both qualitative and quantitative studies, however no qualitative studies met the inclusion criteria. We have made changes to our manuscript to highlight that no qualitative research met our inclusion criteria.

Specific changes to the manuscript are as follows:

Methods, Inclusion criteria: “For inclusion in the review, studies had to: (a) be a primary empirical paper of any study type (e.g., quantitative, qualitative, mixed methods)”.

Results: “All studies that met the inclusion criteria and thus were included in the review were quantitative”.

Narrative synthesis, Study characteristics: “All studies were quantitative;”.

Regarding methodological quality assessment scores for studies on suicidal ideation and behaviours, the mean score across studies was 5.78 ± 2.05 (range 1-10). The quality assessment ratings in the review were not used to determine whether studies were included in the final synthesis, but they were used to aid interpretation of the study findings. However, when comparing quality assessment scores of studies from low quality (≤ 2; e.g., Kwon et al., 2022; McKay et al., 2019; Okado et al., 2021) to excellent/ very good quality (≥ 9; e.g., Chu et al., 2015; Claudius & Axeen, 2022; Marengo et al., 2022) there was no clear pattern among significant findings between low and excellent quality assessment scores. We have added a couple of sentences in the manuscript regarding this. Specific changes are as follows:

*Quality assessment*: “Higher scores indicated a lower likelihood of methodological bias and were used to aid interpretation of the study findings. The first author conducted the quality assessment, and a member of the research team assessed 20% of the methodological quality scores for inter-rater reliability. Any disagreements in scores were resolved via discussion with 100% post-discussion concordance. When comparing studies rated low quality (≤ 2; e.g., Kwon et al., 2022; McKay et al., 2019; Okado et al., 2021) vs excellent/ very good quality (≥ 9; e.g., Chu et al., 2015; Claudius & Axeen, 2022; Marengo et al., 2022), there was no evidence of an association between study quality and the nature or significance of the findings.

*Reviewer: This review aimed to synthesize findings from existing research that have examined risk or protective factors in young people that distinguish between those who have thoughts of self-harm or suicide and those who act on their thoughts. Therefore, what was the outcome? either risk /protective / both? If the outcome was both a risk and protective factor, address each separately.*

*Your discussion is too shallow, so discuss it in more depth. Discuss your study with a systematic review and meta-analysis. Are there differences? If so, why? Compare your independent predictors with other studies. Discuss in more depth. You compared your study to a single cross-sectional study in the discussion section. Is that appropriate? Please revise it. Generally, your comparisons in the discussion part were shallow. So revise it and put your impression/insight based on your findings.*

*Thank you once again for the opportunity to review this work.*

**Response:** Thank you for your comments. The relevant responses are included within distinct subheadings below:

*Outcomes of the review:*

Regarding the outcomes of the review, for the most part, the findings of the review offer support for risk factors for suicidal thoughts to suicidal behaviour, rather than protective factors. However, it is important not to over interpret the conclusion with respect to protective factors. Relatively few studies focused on protective factors, so we do not want to conclude that the absence of evidence suggests evidence of absence. Indeed, future research is needed to determine whether protective factors significantly distinguish between ideation and behaviour groups. We have noted this in the discussion section. Specific changes are as follows:

“Our review found that some protective factors including parental factors (e.g., parental connectedness) and academic achievement distinguished between suicidal ideation and suicide attempts (Okado et al., 2021; Taliaferro & Muehlenkamp, 2014), whereas we found no evidence for protective factors that distinguished self-harm ideation from self-harm behaviours. Hence, for the most part, findings of the review offer support for a selection of risk factors as important in the transition from suicidal thoughts to suicidal behaviour, rather than protective factors. It is important, however, not to over interpret this finding as relatively few studies focused on protective factors. In this regard, the absence of evidence is not the same as finding that protective factors do not play a role. Future research is needed to determine whether protective factors significantly distinguish between ideation and behaviour groups”.

*Discussion*:

In addition to the reviewers’ comments, we have revised our discussion section as suggested. We have compared our study findings with an existing meta-analysis in adults (May & Klonsky, 2016) and note how our findings align and contrast with the findings from this meta-analytical review. We also provide potential explanations for these differences. That said, we agree that the Bayliss et al. (2022) review should be included in the discussion section and have amended the manuscript as such.

Examples of our reference to previous reviews and May and Klonsky’s review from the discussion are provided below:

Discussion, *paragraph* 2: “This finding aligns with a meta-analytical review which found self-harm ideation and behaviours to significantly predict suicide attempts and deaths over time…”.

Discussion, *paragraph* 3: This finding aligns with May and Klonsky’s meta-analytic review [20] in adults where both drug use disorder and sexual abuse distinguished suicide attempts from suicidal ideation, albeit with modest effect sizes.

Discussion, *paragraph* 3: A departure to May and Klonsky’s review [20], however, is that in the current review, physical and emotional abuse appeared to be a risk factor for suicide attempts in young people, whereas alcohol use did not.

We have also revised our manuscript to address the findings from Bayliss et al.’s systematic review. Specific changes are as follows:

Discussion, *paragraph* 2: “Consistent with this, while only examined in two studies, Bayliss et al. [19] found interoceptive deficits to distinguish suicide attempts from suicidal ideation”.

Discussion, *paragraph* 3: “Likewise, this finding also aligns with Bayliss et al.’s systematic review [19] that found painful and provocative events (e.g., abuse, maltreatment) to significantly distinguish suicide attempts from suicidal ideation in adults”.

As suggested, we have compared our independent predictors with other studies, predominantly meta-analyses of risk factors for suicide where available (e.g., Ribeiro et al., 2016, Ribeiro et al., 2018).

Regarding the comment that we compared our study to a single cross-sectional study in the discussion section, we assume this is regarding Wetherall et al.’s (2018) study which examined exposure to self-harm or suicidal behaviours in family and peers. We discuss this study as it was included in our review, however we agree that we shouldn’t give so much weight to the findings of this one cross-sectional study. Hence, we have revised the discussion section regarding this. Specific changes are as follows:

*Discussion*: “While one study included in this review found that exposure to suicide in friends (but not family) was significantly associated with suicide attempts [Wetherall et al., 2018], no consistent patterns emerged across studies in the review. Prior research suggests that exposure to self-harm or suicide in friends may play a more important role in young people who tend to be greatly influenced by their peers and are prone to social contagion”.

**Comments from Reviewer 2:**

Thank you for the opportunity to review this manuscript. Below are the comments on my review:

1. The title is no clearly reflects the subject matter of the article. It needs minor revision to make the object of study of the research clear.

**Response:** Thank you for your comment. We have made minor revisions to the title as recommended. Specific changes are as follows:

“Exploring risk and protective factors which distinguish suicidal and self-harm behaviours from suicidal and self-harm ideation in young people: A systematic review”.

2. In the abstract, clearly present your findings within each subsection.

**Response:** Thank you for your comment. We have now provided subsections in the abstract.

3. In the Introduction section, please cite a reference for line 64. Additionally, this section should define self-harm, suicide attempt, and other suicidal behaviors such as suicide ideation.

**Response:** We have now included a reference for line 64. We have previously defined self-harm and suicide attempts, but we agree that definitions for other suicidal behaviours (e.g., suicidal ideation) are warranted. We have now included definitions for self-harm ideation and suicidal ideation. Specific changes are as follows:

Introduction, *paragraph 1*:

“Despite challenges in defining self-harm and suicide attempts, self-harm can be broadly defined as self-injurious behaviour irrespective of motive; self-harm is a broad term that can include non-suicidal self-injury (self-injurious behaviour without intent to die) [1, 2].Whereas, self-harm ideation depicts thoughts of self-harm without self-injurious behaviour [2]. Suicide attempts are defined as self-injurious, non-fatal behaviours with at least some evidence of intent to die [1]. Conversely, suicidal ideation is defined as thoughts of ending one’s life, where the risk of suicide can progress from relatively passive thoughts to more active thoughts [1]”.

4. Lines 71-75 describe a research gap and your current study's solution. Given that your study is descriptive and does not involve primary research or meta-analysis, the approach to addressing this gap should be clearly articulated. Furthermore, it is recommended to moving these sentences to the concluding section of the introduction.

**Response:** We address a gap in the literature by being the first review to narratively synthesize risk and protective factors that distinguish suicide attempts from suicidal thoughts and self-harm behaviours from thoughts of self-harm or examine factors involved in the transition from thoughts to behaviours over time. Using a narrative synthesis approach, studies were grouped by their outcome variable (e.g., self-harm or suicide) and then by risk and protective factors. We agree and accordingly have moved these sentences to the concluding section of the introduction.

5. Paragraph 2 line 77-87 needs revision and revises it based on scientific study

**Response:** Thank you for your comment. We agree that these sentences require more empirical evidence, in addition to theory, to back up these statements. We have therefore revised this. Specific changes are as follows:

Introduction, *paragraph 2*: “One possible reason for this it that many common risk factors for suicide did not distinguish between risk factors for suicidal ideation and risk factors involved in the transition from suicidal ideation to suicide attempts (see May & Klonsky, 2016). For instance, evidence suggests that many previously identified risk factors for suicide (e.g., depression, hopelessness) are risk factors for suicidal ideation, but not attempts and/or do not significantly distinguish between those with thoughts of suicide and those who attempt suicide [e.g., Mars et al., 2019a; Stewart et al., 2017; Qiu et al., 2017]. The distinction between the development of suicidal ideation and the transition from suicidal ideation to suicide attempts is now widely accepted among researchers and is incorporated into current theoretical models of suicidal behaviour [Klonsky & May, 2014; Klonsky et al., 2018]”.

6. Superficial literature review: The introduction does not critically evaluate existing studies or highlight gaps in the literature, particularly in low- and middle-income countries.

**Response:** Thank you for your comment. We have revised our introduction to critically evaluate existing studies and highlight gaps in the literature. The comment regarding low-and middle-income countries is an important one. Following this comment, we identified only a handful of studies included in our review that were in low and middle-income countries (e.g., Brazil, Iran, Lebanon, Ethiopia, Thailand; Organisation for Economic Co-operation and Development, 2025). We have acknowledged this in the limitations section of the review. Specific changes are as follows:

Limitations of the review and future directions: “In addition, only a few studies included in the review recruited samples from low and middle-income countries (e.g., Ahmadboukani et al., 2023; Dadras & Wang, 2023; Fekadu et al., 2016; Puangsri & Ninla-aesong, 2021; Santana et al., 2016). Given that three quarters of the world’s suicides occur in low and middle-income countries and that the greatest burden of self-harm is felt in low-and-middle income countries (Lemmi et al., 2016; Moran et al., 2024), future research examining risk and protective factors that distinguish self-harm behaviours from self-harm ideation or suicide attempts from suicidal ideation in low and middle-income countries is warranted”.

7. Lack of theoretical framework: The rationale for selecting specific independent variables (e.g., gender difference, and other mental illness that causes suicide Behaviors) is not well-explained.

**Response:** Thank you for your comment. Regarding a theoretical framework, our review was guided by the ideation-to-action theoretical framework (see Klonsky & May, 2014; Van Orden et al., 2010; O’Connor, 2011; Klonsky et al., 2015). For this framework, there are well established variables that have been found to play a role in distinguishing suicide attempts from suicidal ideation/ that are involved in the transition from thoughts to attempts. For example, these include acquired capability (i.e., fearlessness of death and greater tolerance of physical pain, exposure to self-harm/ suicide in others and access to means; Van Orden et al., 2010; O’Connor & Kirtley, 2018) and impulsivity. In our review, we were interested in any factors/ variables (e.g., social, psychological, neural correlates, biological markers) that have been examined within an ideation-to-action framework as we did not want to overlook any novel risk/ protective factors. As a result, we adopted an inclusive approach to examining risk/ protective factors.

8. For the limitations section, you must be more realistic.

**Response:** Thank you for your constructive feedback. We have revised our limitations section in light of your comment, though we weren’t quite sure what you meant precisely. As mentioned above, we have now included reference to the lack of studies included in the review that are in low and middle-income countries.

9. Finally, it is a very important, informative, innovative, and insightful study filling the lacuna from your part of the world.

**Response:** Thank you very much. We are glad you find merit in our review.

**References**

Ati, N. A., Paraswati, M. D., & Windarwati, H. D. (2021). What are the risk factors and protective factors of suicidal behavior in adolescents? A systematic review. *Journal of Child and Adolescent Psychiatric Nursing, 34*(1), 7-18. <https://doi.org/10.1111/jcap.12295>

Auerbach, R.P., Millner, A. J., Stewart, J. G., & Esposito, E. C. (2015). Identifying differences between depressed adolescent suicide ideators and attempters. *Journal of Affective Disorders, 186,* 127-133. <https://doi.org/10.1016/j.jad.2015.06.031>

Bayliss, L. T., Christensen, S., Lamont-Mills, A., & du Plessis, C. (2022). Suicide capability within the ideation-to-action framework: A systematic scoping review. *PloS one*, *17*(10), e0276070. <https://doi.org/10.1371/journal.pone.0276070>

Chu, C., Buchman-Schmitt, J. M., Joiner, T. E. (2015). Autobiographical memory perspectives in task and suicide attempt recall: A study of young adults with and without symptoms of suicidality. *Cognitive Therapy and Research, 39,* 766-775. <https://doi.org10.1007/s10608-015-9704-6>

Claudius, I., Axeen, S. (2022). Differences in comorbidities between children and youth with suicide attempts versus ideation presenting to the emergency department. *Archives of Suicide Research, 26,* 280-289. <https://doi.org/10.1080/13811118.2020.1795017>

Fekadu, A., Medhin, G., Selamu, M., Shiferaw, T., Hailemariam, M., Rathod, S.D. et al.

(2016). Non-fatal suicidal behaviour in rural Ethiopia: A cross-sectional facility-and

population-based study. *BMC Psychiatry, 16,* 1-9.

<https://doi.org/10.1186/s12888-016-0784-y>

Hawton, K., Saunders, K.E., O'Connor, R. C. (Self-harm and suicide in adolescents. *The Lancet*, *379*(9834), 2373-2382. <https://doi.org/10.1016/S0140-6736(12)60322-5>

Klonsky, E. D., & May, A. M. (2014). Differentiating suicide attempters from suicide ideators: A critical frontier for suicidology research. *Suicide and Life‐Threatening Behavior*, *44*(1), 1-5. <https://doi.org/10.1111/sltb.12068>

Klonsky D. E., May, A. M. The Three-Step Theory (3ST): A new theory of

suicide rooted in the “ideation-to-action” framework. (2015). *International Journal of Cognitive Therapy, 8,* 114-129. <https://doi.org/10.1521/ijct.2015.8.2.114>

Kwon, M., Kim, S. A., Seo, K. (2022). Factors influencing suicide attempts of adolescents with suicidal thoughts in South Korea: Using the 15th Korean Youth Risk Behavior Web-Based Survey (KYRBS). *Iranian Journal of Public Health, 51*(9), 1990-1998. <https://doi.org/10.18502/ijph.v51i9.10553>

Lemmi, V., Bantjes, J., Coast, E., Channer, K., Leone, T., McDaid, D., ... & Lund, C. (2016). Suicide and poverty in low-income and middle-income countries: A systematic review. *The Lancet Psychiatry, 3*(8), 774-783. https://doi.org/[10.1016/S2215-0366(16)30066-9](https://doi.org/10.1016/S2215-0366(16)30066-9)

Mars, B., Heron, J., Klonsky, E. D., Moran, P., O'Connor, R. C., Tilling, K., Wilkinson, P., & Gunnell, D. (2019). Predictors of future suicide attempt among adolescents with suicidal thoughts or non-suicidal self-harm: a population-based birth cohort study. *The Lancet Psychiatry, 6*(4), 327-37. <https://doi.org/10.1016/S2215-0366(19)30030-6>

Marengo, L., Douaihy, A., Zhong, Y., Krancevich, K., Brummit, B., Sakolsky, D. et al. (2022). Opioid use as a proximal risk factor for suicidal behavior in young adults. *Suicide and Life-Threatening Behavior, 52,* 199-213. <https://doi.org/10.1111/sltb.12806>

May, A. M., & Klonsky, E. D. (2016). What distinguishes suicide attempters from suicide ideators? A meta‐analysis of potential factors. *Clinical Psychology: Science and Practice*, *23*(1), 5-20. <https://doi.org/10.1111/cpsp.12136>

McKay, T., Berzofsky, M., Landwehr, J., Hsieh, P., Smith, A. (2019). Suicide etiology in youth: Differences and similarities by sexual and gender minority status. *Children and Youth Services Review, 102,* 79-90. <https://doi.org/10.1016/j.childyouth.2019.03.039>

Moran, P., Chandler, A., Dudgeon, P., Kirtley, O. J., Knipe, D., Pirkis, J., ... & Christensen, H. (2024). The Lancet Commission on self-harm. *The Lancet, 404*(10461), 1445-1492. <https://doi.org/10.1016/S0140-6736(24)01121-8>

O'Connor, R. C. (2011). Towards an integrated motivational–volitional model of suicidal behaviour. International handbook of suicide prevention: *Research, Policy and Practice,* 181-198.

O'Connor, R. C., & Kirtley, O. J. (2018). The integrated motivational–volitional model of suicidal behaviour. *Philosophical Transactions of the Royal Society B: Biological Sciences*, *373*(1754), 20170268. <https://doi.org/10.1098/rstb.2017.0268>

Office for National Statistics (2024). Suicides in England and Wales: 2023 registrations. <https://www.ons.gov.uk/peoplepopulationandcommunity/birthsdeathsandmarriages/deaths/bulletins/suicidesintheunitedkingdom/2023> [Accessed 15th April 2025].

Okado, I., Floyd, F. J., Goebert, D., Sugimoto-Matsuda, J., & Hayashi, K. (2021). Applying ideation-to-action theories to predict suicidal behavior among adolescents. *Journal of Affective Disorders, 295,* 1292-1300. <https://doi.org/10.1016/j.jad.2021.08.137>

Organisation for Economic Co-operation and Development (2025). ODA recipients: countries, territories, and international organisations. <https://www.oecd.org/en/topics/sub-issues/oda-eligibility-and-conditions/dac-list-of-oda-recipients.html> [Accessed 22nd April 2025].

Qiu, T., Klonsky, E. D., & Klein, D. N. (2017). Hopelessness predicts suicide ideation but not attempts: A 10‐year longitudinal study. *Suicide and Life‐Threatening Behavior, 47*(6), 718-722. <https://doi.org/10.1111/sltb.12328>

Ribeiro, J. D., Franklin, J. C., Fox, K. R, Bentley, K. H, Kleiman, E. M., Chang, B. P, & Nock, M. K (2016). Self-injurious thoughts and behaviors as risk factors for future suicide ideation, attempts, and death: a meta-analysis of longitudinal studies. *Psychological Medicine, 46*(2): 225-236. <https://doi.org/10.1017/S0033291715001804>

Ribeiro, J. D., Huang X., Fox K. R., Franklin J. C. (2018). Depression and hopelessness as risk factors for suicide ideation, attempts and death: Meta-analysis of longitudinal studies. *British Journal of Psychiatry, 212*, 279–286. <https://doi.org/10.1192/bjp.2018.27>

Rodway, C., Tham, S. G., Ibrahim, S., Turnbull, P., Kapur, N., & Appleby, L. (2020). Children and young people who die by suicide: childhood-related antecedents, gender differences and service contact. *BJPsych Open*, *6*(3), e49. <https://doi.org/10.1192/bjo.2020.33>

Shaffer, D., Gould, M. S., Fisher, P., Trautman, P., Moreau, D., Kleinman, K., & Flory, M. (1996). Psychiatric diagnosis in child and adolescent suicide. *Archive of General Psychiatry, 53,* 339–348. <https://doi.org/10.1001/archpsyc.1996.01830040075012>

Stewart, J. G., Shields, G. S., Esposito, E. C., Cosby, E. A., Allen, N. B., & Slavich, G. M. et al. (2019). Life stress and suicide in adolescents. *Journal of Abnormal Child Psychology, 47,* 1707-1722. <https://doi.org/10.1007/s10802-019-00534-5>

Taliaferro, L. A., & Muehlenkamp, J. J. (2014). Risk and protective factors that distinguish adolescents who attempt suicide from those who only consider suicide in the past year. *Suicide and Life‐Threatening Behavior, 44*(1), 6-22. <https://doi.org/10.1111/sltb.12046>

Van Orden, K. A., Witte, T. K., Cukrowicz, K. C., Braithwaite, S. R., Selby, E. A., & Joiner

Jr, T. E. (2010). The interpersonal theory of suicide. *Psychological Review, 117*, 575-600.

<https://doi.org/10.1037/a0018697>

Wang, Y., Liu, J., Chen, S., Zheng, C., Zou, X., Zhou, Y. (2024). Exploring risk factors and their differences on suicidal ideation and suicide attempts among depressed adolescents based on decision tree model. *Journal of Affective Disorders, 352,* 87-100. [https://doi.org/10.1016/j.jad.2024.02.035](https://doi.org/10.1016/j.jad.2024.02.035)

Wetherall, K., Cleare, S., Eschle, S., Ferguson, E., O'Connor, D. B., O'Carroll, R. E. et al. (2018). From ideation to action: Differentiating between those who think about suicide and those who attempt suicide in a national study of young adults. *Journal of Affective Disorders. 241,* 475-483. <https://doi.org/10.1016/j.jad.2018.07.074>

World Health Organization. (2024). Adolescent and young adult health. https://www.who.int/news-room/fact-sheets/detail/adolescents-health-risks-and-solutions [Accessed 6^th^ May 2025].
